# Gelation and Retrogradation Mechanism of Wheat Amylose

**DOI:** 10.3390/ma4101763

**Published:** 2011-10-10

**Authors:** Yukihiro Tamaki, Teruko Konishi, Masakuni Tako

**Affiliations:** Department of Subtropical Bioscience and Biotechnology, University of the Ryukyus, Nishihara, Okinawa 903-0213, Japan; E-Mails: shihakugyoku@hotmail.com (Y.T.); konishi@agr.u-ryukyu.ac.jp (T.K.)

**Keywords:** wheat amylose, gelation mechanism, hydrogen bonding, intra- andintermolecular association

## Abstract

The flow behavior, dynamic viscoelasticity, and optical rotation of aqueous solutions of wheat amylose were measured using a rheogoniometer and a polarimeter. The amylose solutions, at 25 °C, showed shear-thinning behavior at a concentration of 1.2%, but plastic behavior at 1.4 and 1.6%, the yield values of which were estimated to be 0.6 and 1.0 Pa, respectively. The viscosity of the wheat amylose increased a little with increase in temperature up to 10 or 20 °C at 1.2% or 1.4 and 1.6%, which was estimated to be a transition temperature. The elastic modulus increased with increase in concentration, and increased with increasing temperature up to 20, 25 and 30 °C, which was estimated to be a transition temperature, respectively, then decreased gradually but stayed at a large value even at high temperature (80 °C). A very low elastic modulus of the wheat amylose was observed upon addition of urea (4.0 m) and in alkaline solution (0.05 m NaOH) even at low temperature. The optical rotation of wheat amylose solution increased a little with decreasing temperature down to 25 °C, then increased rapidly with further decrease in the temperature. The mode of gelation mechanism of amylose molecules, which was previously proposed, was confirmed and a retrogradation mechanism of wheat amylose was proposed.

## 1. Introduction

**Scheme 1:**
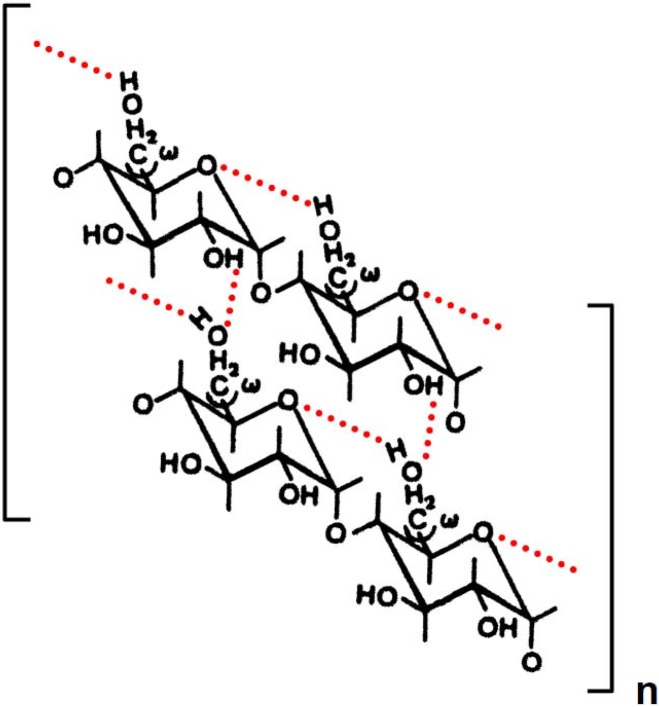
Possible mode of intra- and intermolecular hydrogen bonding of potato amylose in aqueous solution. Dotted lines represent hydrogen bonding. The conformational changes in the interaction between neighboring OH-6 of the d-glucosyl residues are expressed in terms of the angles of rotation, ω. The model corresponds to a double stranded helix. A tertiary structure of the wheat amylose molecules may consist of two identical left handed, 6-fold helices in aqueous solution as in solid state.

Starch is a major component of many food plants and is used in food, cosmetics, paper, textile, and some other industries as thickening, stabilizing, or gelling (pasting) agents. Starch consists of two polymers, amylose and amylopectin, the molar ratio of which is about 20–30% and 80–70%, respectively. Amylose is a linear polysaccharide composed of 1,4-linked α-d-glucosyl residues by definition, but the actual specimens, which are isolated and purified from starches, include slightly branched molecules [1,2,3]. Amylose produces a gel by cooling after heating aqueous suspension at high temperature range (120–140 °C). We have proposed a possible gelation mechanism of potato amylose in aqueous solutions [4]. An intramolecular hydrogen bonding takes place between OH-6 and the adjacent hemiacetal oxygen atom of the d-glucopyranosyl residues, as illustrated in Scheme 1. In addition, intermolecular hydrogen bonding takes place between OH-2 and the adjacent O-6 of the d-glucopyranosyl residues on different molecules. A part of the intermolecular hydrogen bonding, side-by-side association, breaks down above a transition temperature, 25–35 °C, during increase in the temperature under a steady shearing force. Residual intermolecular and intramolecular hydrogen bondings are lost above another transition temperature, 80–90 °C. Under steady angular force, however, both intra- and intermolecular hydrogen bondings of amylose molecules are stable until the temperature reached 65–80 °C. Although this model corresponds to a double-stranded helix, a single helix also seems to exist in part with the formation of side-by-side intermolecular hydrogen bondings in aqueous solution where C-3 of OH groups of d-glucosyl residues take part in the interaction.

We have discussed the molecular origin for the thermal stability of rice amylopectin, which is a branched macromolecule composed of (1→4)- and (1→6)-α-d-glucan chains in aqueous solution and concluded that the molecules are involved in intramolecular hydrogen bonding and van der Waals forces of attraction [5,6,7]. Intramolecular hydrogen bonding, together with van der Waals forces of attraction played a dominant role in the thermal stability for viscosity and dynamic viscoelasticity of rice amylopectin in aqueous solution. Long chains of rice amylopectin molecules (B2-3) might be involved in intramolecular associations. We have reported that rheological properties of potato [8] and wheat amylopectin in aqueous solution [9,10]. The wheat amylopectin molecules are involved in compacted conformation which are caused by long chains B2-3 where about 40% of B chains carried no A chains [11]. This suggested that there were intramolecular hydrogen bonding on the long chains to make compacted conformation. We have concluded that the structure of wheat amylopectin molecules seems to be different from that of rice amylopectin.

**Scheme 2:**
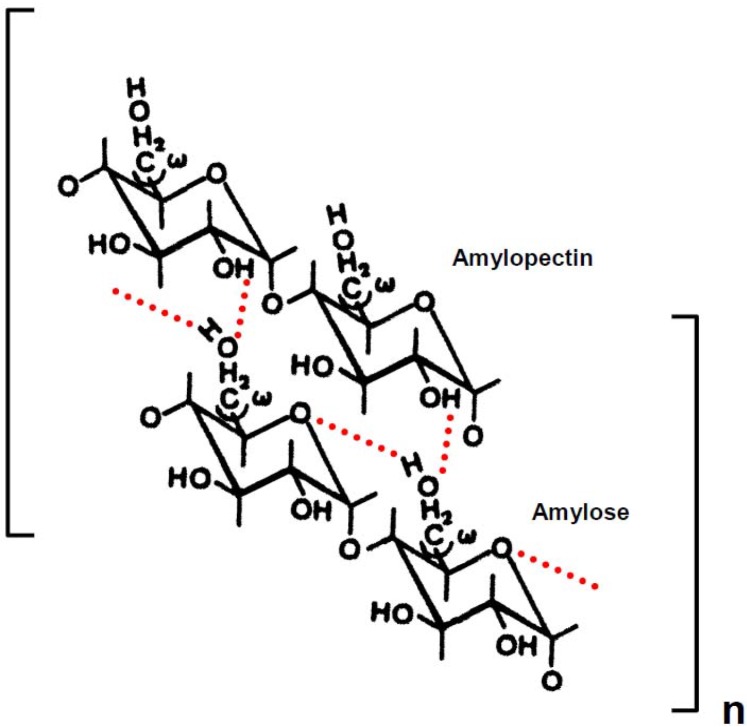
Possible mode of gelatinization mechanism of rice, potato and wheat starch in aqueous solution. Dotted lines represent hydrogen bonding. The chains of amylopectin are short side-chains (A or B1).

**Scheme 3:**
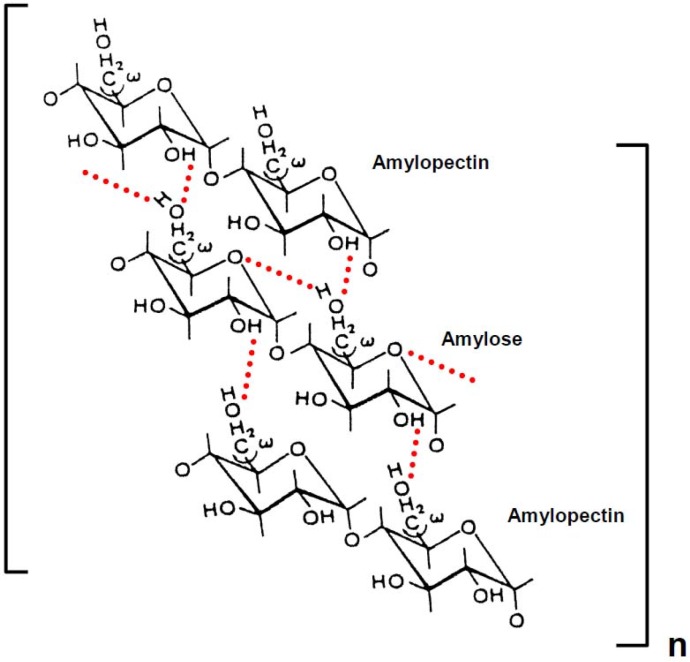
Possible mode of retrogradation mechanism of rice, potato and wheat starch in aqueous solution. Dotted lines represent hydrogen bonding. The chains of amylopectin are short side-chains (A or B1).

We have proposed gelatinization and retrogradation mechanisms of rice [12,13], potato [14] and wheat [15,16] starches in aqueous solutions, as illustrated in Scheme 2 and Scheme 3. An intermolecular hydrogen bonding of rice, potato and wheat starch might take place between O-6 of the amylose and OH-2 of the amylopectin molecules. The short amylopectin side-chains (A and B1), which are not involved in intramolecular hydrogen bonding, may take part in the intermolecular associations. Intermolecular hydrogen bonding between amylose and amylopectin is thermally stable. The intermolecular hydrogen bonding, together with intramolecular association within long chains (B2−4) of amylopectin molecules, dissociate above the transition temperature in solutions of 4.0 m urea and 0.05 m NaOH. The dynamic viscoelasticity increased when rice, potato and wheat starch solutions were stored at 25 °C and 4 °C for 24 h. We have concluded that, after formation of intermolecular hydrogen bonding between O-6 of the amylose and OH-2 of the amylopectin molecules, another intermolecular hydrogen bonding may form between OH-2 of a d-glucose residue of the former molecule and O-6 of a short side chain (A and B1) of the latter molecule (Scheme 3). Two or more short side-chains (A and B1) of an amylopectin molecule may associate with an amylose molecule. After saturation of intermolecular hydrogen bonding between amylose and amylopectin molecules, an intermolecular association also takes place between amylopectin molecules through hydrogen bonding (Scheme 3), because the molar ratio of amylose to amylopectin was 1:5 [17,18,19,20,21,22,23,24]. A side-by-side association between O-3 and OH-3 of d-glucosyl residue on different amylopectin molecules may also take place (Scheme 3).

In the present study, we analyze the rheological behavior of a solution of wheat amylose with respect to its association characteristics in comparison with solutions of potato amylose.

## 2. Results and Discussion

### 2.1. Chemical Characteristics of Wheat Amylose

The number-average degree of polymerization (DPn) of purified wheat amylose was estimated to be 250, with a molecular mass of 40,500, based on the number of glucosyl units per reducing residue. The degree of polymerization of the wheat amylose is a little smaller than that of potato amylose (300; Sigma Chemical Co., Ltd.) [4].

### 2.2. Flow Behavior

**Figure 1:**
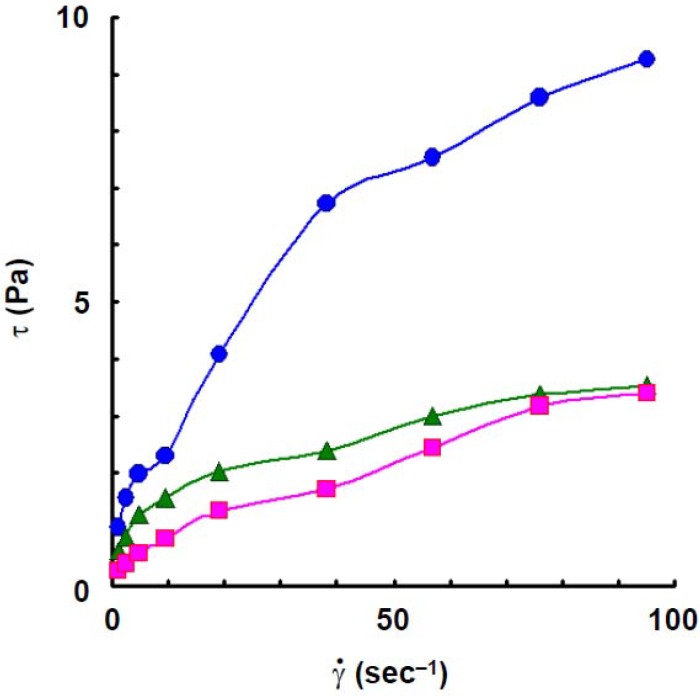
Flow curves, at 25 °C, of wheat amylose at various concentrations. Concentrations: 1.2%, 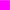; 1.4%, 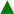; 1.6%, 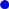.

To compare the rheological behaviors of wheat amylose to those of potato amylose, the viscosity and dynamic viscoelasticity were measured under the same conditions as those of the latter [4]. The flow curves, at 25 °C, of wheat amylose aqueous solution at various concentrations are shown in Figure 1. Though the flow curve at a concentration of 1.2% solution of wheat amylose approximated shear-thinning behavior, it showed plastic behavior and the yield value was estimated to be 0.6 Pa at 1.4%. Plastic behavior was also observed in 1.6% solution and the yield value increased a little (1.0 Pa). The yield value indicates that a secondary association is involved within or between wheat amylose molecules in aqueous solution [4,25,26,27,28,29]. As reported previously [4], a curious flow behavior was observed in a potato amylose solution at 1.6%: the shear stress decreased rapidly with an increase of shear rate up to 9.5 s^−1^, then it increased gradually with further increasing shear rate. The phenomenon, showing a decrease of shear stress with increasing shear rate up to 9.5 s^−1^, might be caused by a rapid breakdown of an intermolecular association of the molecules.

### 2.3. Viscosity

**Figure 2:**
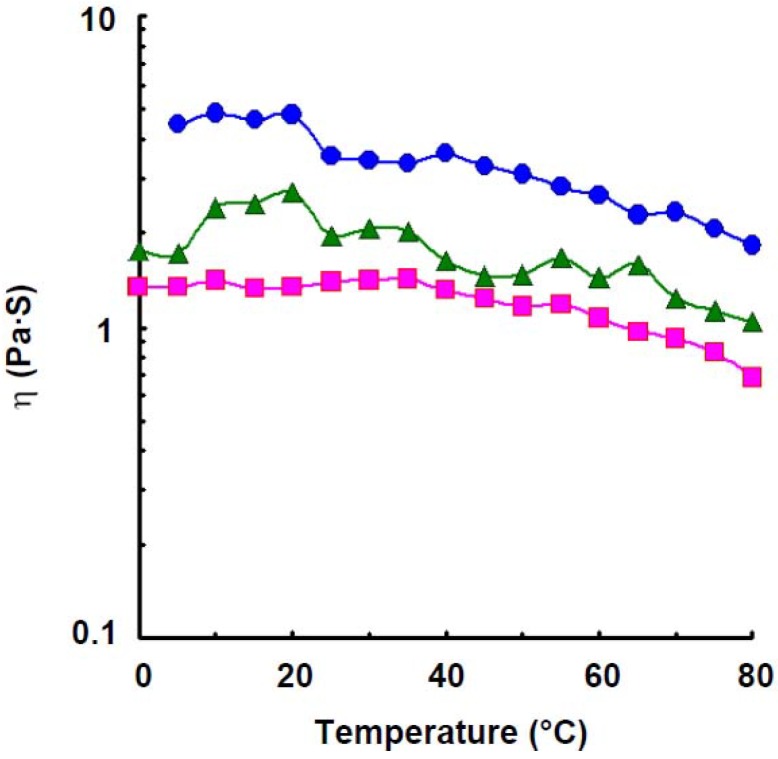
Effect of temperature on the viscosity of wheat amylose at various concentrations at 9.5 s^−1^. Concentration: 1.2%, 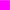; 1.4%, 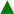; 1.6%, 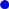.

As shown in Figure 2, the apparent viscosity of amylose solution at a concentration of 1.2% increased a little with increasing temperature up to 35 °C which was estimated to be a transition temperature, and decreased a little with further increase in temperature, but retained large values even at 70 °C which was estimated to be secondary transition temperature. Though the viscosity stayed at constant value with increase in temperature up to 5 °C, it increased rapidly with further increase in the temperature to 10 °C, the increase in the viscosity was observed up to 20 °C, which was estimated to be a transition temperature in a solution of 1.4%. The viscosity, however, decreased a little with further increase in temperature but showed large value even at 65 °C which was estimated to be secondary transition temperature, then decreased rapidly. For 1.6% solution, the viscosity increased a little with increase in temperature up to 20 °C which was estimated to be a transition temperature, then decreased rapidly with increasing temperature to 25 °C, but stayed at a constant value up to 40 °C, then decreased a little with further increase in the temperature and retained large values even at 70 °C which was estimated to be secondary transition temperature, then decreased gradually. A curious viscosity profile was observed in wheat amylose solution. The phenomenon showing transition temperature was also observed in κ-carrageenan [25], ι-carrageenan [26], agarose [27], gellan gum [28], deacetylated rhamsan gum [29], and potato amylose [4] solutions. Particularly, curious viscosity profile was also observed in the last named solution where it decreased rapidly when the temperature reached 25, 30, and 35 °C, at 1.2, 1.4, and 1.6%, these temperatures estimated to be first transition temperatures. However, after reaching the first transition temperature, the amylose solutions essentially maintained a constant viscosity up to 80, 90, and 90 °C, respectively, which were estimated to be second transition temperature. The results indicated that there were two stepwise conformational transitions in potato amylose molecules under shearing force over a temperature range of 25–35 °C and of 80–90 °C, respectively, where a part of hydrogen bonding dissociated above the first transition temperature and most of hydrogen bonding dissociated above the second transition temperature. Accordingly, for wheat amylose, a part of a secondary association dissociates above the first transition temperature, 20 °C and the residual association is stable even at 65–70 °C, then dissociates again with further increase in temperature.

### 2.4. Dynamic Viscoelasticity

**Figure 3:**
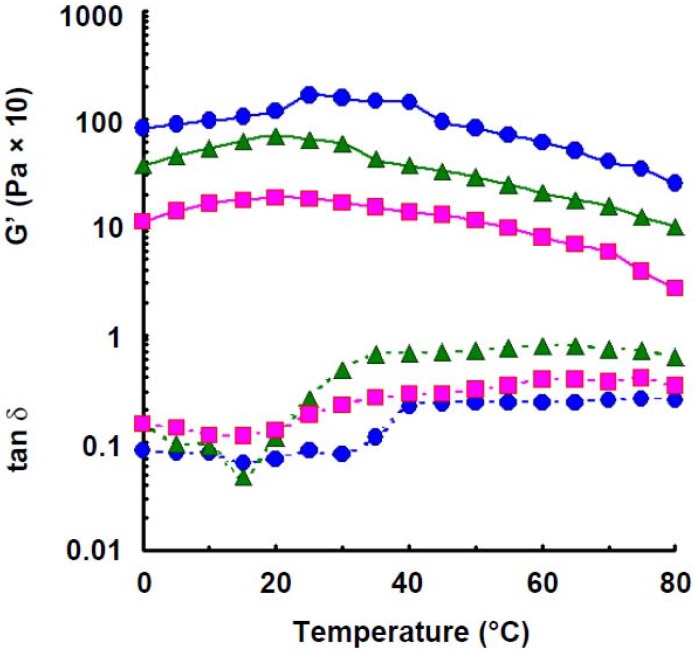
Effect of temperature on the elastic modulus and tan δ of wheat amylose at various concentrations. Concentrations: 1.2%, 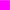; 1.4%, 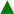; 1.6%, 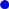. Lines: storage modulus, solid; tan δ, dotted.

Gelation occurred at a concentration above 1.4% wheat amylose solution at a low (0 °C) temperature. Figure 3 shows effect of temperature on elastic modulus of wheat amylose at various concentrations. For 1.2% solution, elastic modulus increased with increase in temperature up to 20 °C, which was estimated to be a transition temperature, and stayed at a constant value up to 40 °C, decreased gradually with further increase in temperature of 70 °C, which was estimated to be a transition temperature, then decreased rapidly. This indicates that there are two stepwise conformational transitions in wheat amylose molecules as in potato amylose molecules in aqueous solution. For 1.4% solution, elastic modulus also increased with increase in temperature up to 20 °C, then decreased gradually with further increase in temperature, but kept a large value even at 80 °C. The elastic modulus of 1.6% solution also increased with increase in temperature up to 25 °C, it kept a constant value, but when the temperature reached 40 °C, it decreased rapidly with further increasing temperature to 45 °C, then decreased gradually with further increase in the temperature, but kept a large value even at 80 °C. On the other hand, tan δ value of wheat amylose solution at 1.2% showed a value of 0.16 at low temperature (0 °C) and decreased a little with increase in temperature up to 15 °C, but it increased rapidly with further increase in temperature to 40 °C, then increased a little. The tan δ at a concentration of 1.4% showed a little lower value (0.14) than that of 1.2% solution and decreased a little with increase in temperature up to 15 °C, but it increased rapidly with increase in temperature to 35 °C and retained a constant value with further increase in temperature. The tan δ of 1.6% solution had a very low value, 0.08 (0 °C), during increase in temperature up to 30 °C, then increased rapidly with further increase in the temperature to 40 °C and kept constant showing a value of 0.24, indicating an intermolecular association has been established tightly even at high temperature range. Wheat amylose molecules in aqueous solution are liable to bind a secondary association with increase in temperature up to 15–20 °C under steady angular force.

**Figure 4:**
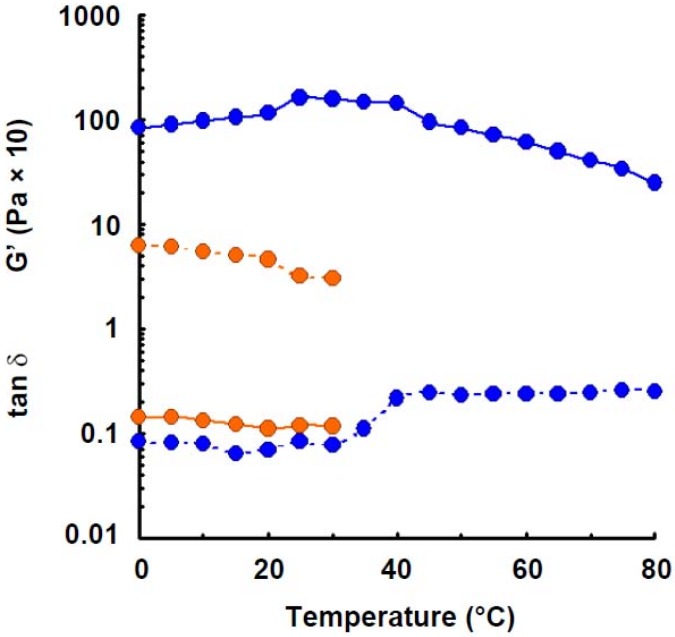
Effect of temperature on the elastic modulus and tan δ of wheat amylose (1.6%) with addition of urea (4.0 m). Symbols: preparation in distilled water, 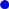; preparation with addition of urea, 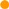. Lines: storage modulus, solid; tan δ, dotted.

Elastic modulus of wheat amylose solution (1.6%) was measured in the presence of urea (4.0 m), which is known as a hydrogen-bonding breaker, as shown in Figure 4. The least elastic modulus was observed. Such phenomenon was also observed in potato amylose [4], agarose [27] and curdlan [30] where hydrogen bonding participated in intra- and intermolecular association in aqueous solution. The result indicates that hydrogen bonding takes part in the gel-formation of wheat amylose molecules in aqueous solution.

**Figure 5:**
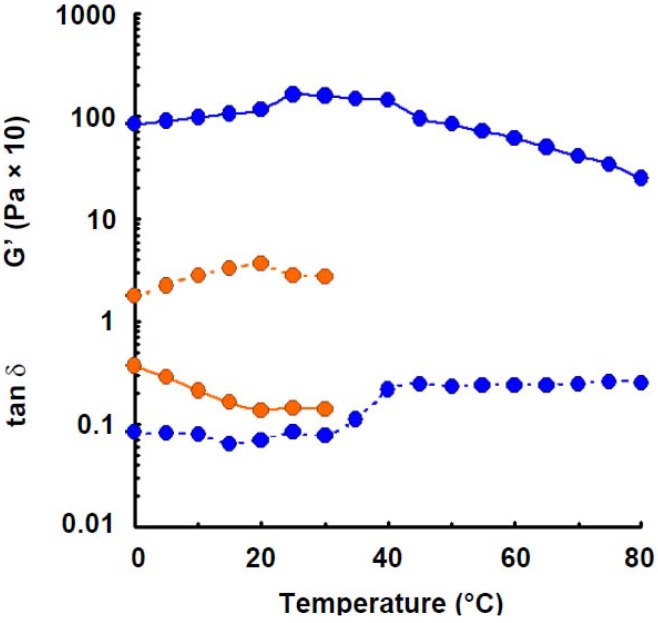
Effect of temperature on the elastic modulus and tan δ of wheat amylose (1.6%) in alkaline solution (0.05 m NaOH). Symbols: preparation in distilled water, 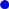; preparation in 0.05 m NaOH, 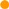. Lines: storage modulus, solid; tan δ, dotted.

Elastic modulus of wheat amylose (1.6%) was also measured in 0.05 m NaOH solution. The least storage modulus was observed, as shown in Figure 5. The result suggests that hydrogen bonding takes part in the gel-formation of wheat amylose molecules in aqueous solution, because NaOH is also a hydrogen-bonding breaker.

### 2.5. Optical Rotation

The optical rotation of a 1.0% solution of wheat amylose at various temperatures was determined after dissolving the sample at 145 °C for 15 min, then cooling the temperature from 60 to 20 °C. The optical rotation increased gradually with decreasing temperature up to 25 °C, then it increased rapidly. This suggests that an intermolecular hydrogen bonding of wheat amylose molecules may take place at a temperature below 25 °C.

**Table 1:** Optical rotation of wheat amylose at 589 nm.

**Temperature (°C)**		60	50	40	30	25	20
**Optical rotation (°)**		+0.827	+0.839	+0.857	+0.883	+0.901	+0.983

## 3. Experimental Section

### 3.1. Materials

A wheat starch was supplied by Sigma Chem. Co., Ltd, U.S.A. and amylose was separated by the method of Lansky *et al.* [31] with minor modification [2]. The wheat starch (10 g) was dissolved in methyl sulfoxide (300 mL) by heating at 100 °C with stirring under nitrogen for 6 h, and ethanol (300 mL) was then added to the solution. The precipitate was collected by centrifugation (10,000 rpm for 20 min). The sample was dispersed in water (300 mL) at 80 °C, and a mixture of 1-butanol (100 mL), 3-methyl-1-butanol (100 mL), and water (1,300 mL) was added. The dispersion was stirred and boiled under reflux for 3 h, cooled to 50 °C, and kept for overnight at room temperature and then stored at 10 °C for 48 h. The precipitate was collected by centrifugation and then suspended in aqueous 10% 1-butanol (500 mL) and boiled under reflux for 6 h under nitrogen, and cooled. After cooling, the precipitate was collected by centrifugation (10,000 rpm for 15 min). The precipitate was suspended in aqueous 10% 1-butanol again. The suspension was boiled under reflux for 1 h, cooled, and stored at room temperature for 24 h. The precipitate was obtained by centrifugation. The amylose specimens (1.6 g) were stored in the wet amylose-1-butanol complex. When the chemical, physical and rheological measurements were performed, the amylose sample was dried *in vacuo*.

### 3.2. Methods

The number-average degree of polymerization (DPn) was calculated on the basis of the reducing and total carbohydrate. The reducing residues were determined by Somogyi [32] and Nelson [33] methods. Total carbohydrate was determined by the phenol-sulfuric acid method [34].

Aqueous solutions were obtained with an autoclave (MC-30321, Ikemoto Rika Kogyo Co., Ltd.) by heating mixtures of amylose and distilled water in sealed flasks at 145 °C for 15 min. Optical rotations were measured at 589 nm with an automatic digital polarimeter DIP-180 (Japan Spectroscopic Co., Ltd), for a solution of 1.0% (W/V) in water, with cooling system.

Viscosity at various shear rates (1.19–95.03 s^−1^) and elastic modulus at a fixed angular velocity (3.77 rads^−1^) were determined with a rheogoniometer (Iwamoto Seisakusho Co., Ltd., Kyoto, Japan) consisting of a coaxial cylinder (1.8 cm diam.) with a rotating outer cylinder (2.2 cm diam.). The temperature was controlled by circulating oil from a thermo-cool instrument (LCH-130F, Toyo Co., Ltd.), over the temperature range of 0–80 °C and raised at a stepwise rate of 1 °C min^−1^. Shear rates (γ), shear stress (τ) and apparent viscosity (η) were calculated with an equation of Margules [35]. Dynamic viscosity (η’) and storage modulus (G’) were calculated by modification of Markovitz’s equation [36]. The loss tangent was calculated from the relationship, tanδ = G’’/G’ where G’’ = ωη’ is the loss modulus, and ω is the angular velocity of the outer cylinder. The measurements were carried out in triplicate.

## 4. Conclusions

The viscosity and elastic modulus of wheat amylose aqueous solutions were essentially in agreement with those of potato amylose [4]. Transition temperature at which viscosity decreased rapidly was observed in aqueous solutions of wheat amylose at 20 °C, but it retained a large value even at 65–70 °C, which was estimated to be secondary transition temperatures. Transition temperature was also observed in elastic modulus at 20–25 °C at various concentrations (1.2, 1.4 and 1.6%). These results support the notion that intramolecular hydrogen bonding occurs in wheat amylose and has a great influence on gelling properties together with intermolecular hydrogen bonding in amylose molecules in aqueous solutions.

Thus, we conclude that an intramolecular hydrogen bonding may take place between OH-6 and the adjacent hemiacetal oxygen atom of the d-glucosyl residues. This bonding is likely due to the flexibility of the α-(1→4)-linkage and extended conformations at high temperatures. In addition, intermolecular hydrogen bonding may take place between OH-2 and the adjacent O-6 of the D-glucosyl residues on different molecules. A part of the intermolecular hydrogen bonding, side-by-side association, breaks down above a transition temperature (20 °C) under shearing force. However, another intermolecular, together with intramolecular hydrogen bonding is stable even at high temperature (80 °C). Under angular velocity, however, both intra- and intermolecular hydrogen bonding of wheat amylose molecules are established tightly with increase in temperature up to 20–25 °C and dissociate a little with further increase in temperature, but stable even at high temperature (80 °C). The associations correspond to a double-stranded helix. Within the double helix, interstrand stabilization is achieved through the intermolecular hydrogen bonding. However, a single helix also seems to exist in part with the formation of side-by-side intermolecular hydrogen bonding, where OH-3 of the d-glucosyl residues may participate in aqueous solution. A tertiary structure of amylose molecules may consist of two identical, left-handed, 6-fold helices in aqueous solution as in solid state [37].

**Scheme 4:**
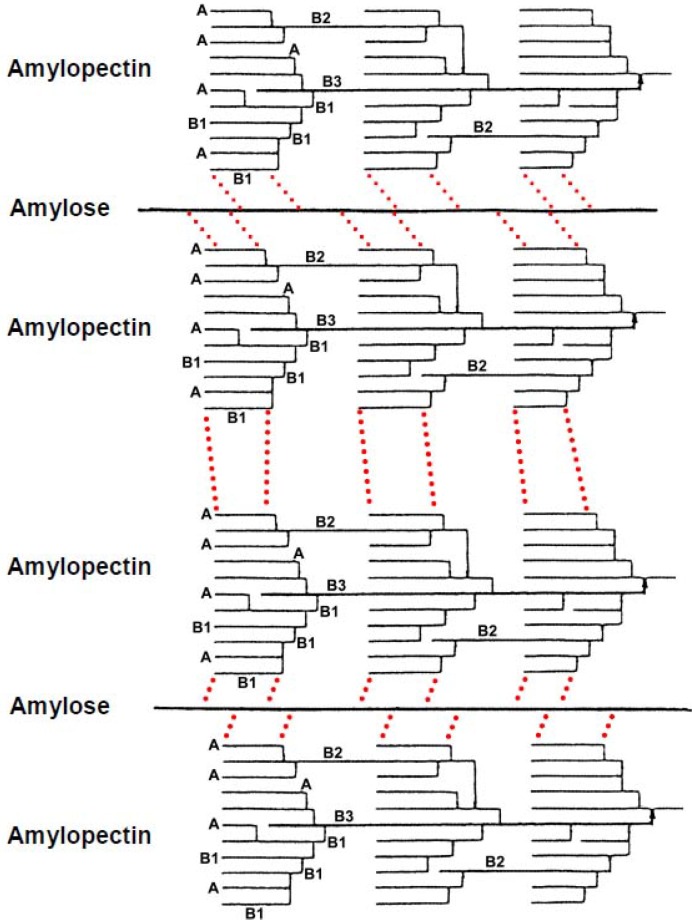
Possible mode of retrogradation mechanism of rice, potato and wheat starch in aqueous solution. Dotted lines represent hydrogen bonding.

**Scheme 5:**
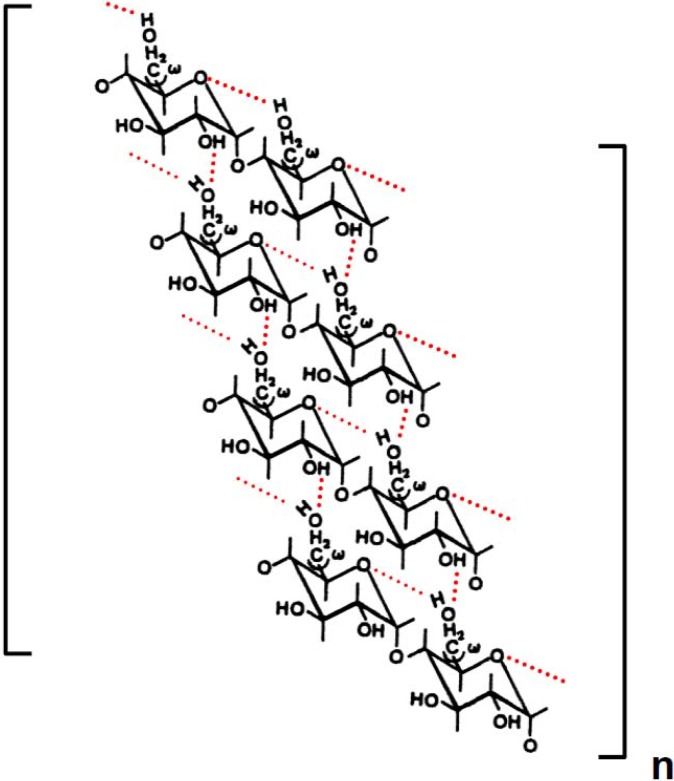
Possible mode of retrogradation mechanism of wheat amylose in aqueous solution. Dotted lines represent hydrogen bonding.

Amylose molecules, however, in aqueous solution are notoriously unstable, and retrogradation results in increase in turbidity and eventually precipitation occurs. Indeed, we have observed such a phenomenon in wheat amylose aqueous solution. Accordingly, the retrogradation seems to occur by shrinkage of the amylose molecules which were caused by decrease of the kinetic energy and Brownian motion of the polymer and water molecules [38] and results in new formation of intramolecular and intermolecular hydrogen bonding within the hemiacetal oxygen atom and the adjacent OH-6 of the d-glucosyl residues and between O-6 and OH-2 of D-glucosyl residues on different molecules, as shown in Scheme 5. At the final stage, side-by-side association between O-3 and OH-3 of d-glucosyl residues on different amylose molecules may also take place with hydrogen bonding. Much more intense intra- and intermolecular hydrogen bonding may result in precipitation of the wheat amylose molecules in aqueous media. This (Scheme 5) and preceding papers concerning amylose (Scheme 1), amylopectin and starches (Scheme 2, Scheme 3, and Scheme 4) may provide useful information and suggestions not only for academic researchers but also for technical scientists.
